# Collaborative care model improves self-care ability, quality of life and cardiac function of patients with chronic heart failure

**DOI:** 10.1590/1414-431X20176355

**Published:** 2017-09-21

**Authors:** C.Y. Hua, Y. Huang, Y.H. Su, J.Y. Bu, H.M. Tao

**Affiliations:** 1 Department of Hematology and Rheumatology, The Fifth Affiliated Hospital of Sun Yat-Sen University, Zhuhai, Guangdong, China; 2 Department of Cardiothoracic Surgery, The Fifth Affiliated Hospital of Sun Yat-Sen University, Zhuhai, Guangdong, China; 3 Department of General Surgery I, The Fifth Affiliated Hospital of Sun Yat-Sen University, Zhuhai, Guangdong, China; 4 Department of Nursing, The Fifth Affiliated Hospital of Sun Yat-Sen University, Zhuhai, Guangdong, China

**Keywords:** Collaborative care model, Chronic heart failure, Self-care, Quality of life, Cardiac function

## Abstract

Chronic heart failure (CHF) is a common chronic disease that requires much care. This study aimed to explore the effects of collaborative care model (CCM) on patients with CHF. A total of 114 CHF patients were enrolled in this study, and were randomly and equally divided into two groups: control and experimental. Patients in the two groups received either usual care or CCM for 3 continuous months. The impacts of CCM on the self-care ability and quality of life were assessed using self-care of heart failure index and short form health survey 12, respectively. Further, cardiac function was assessed by measuring left ventricular ejection fraction (LVEF) and the level of N-terminal pro-B-type natriuretic peptide (NT-proBNP), and by the 6-min walking test. Clinical and demographic characteristics of patients in the control and CCM groups were statistically equivalent. Compared with usual care, CCM significantly enhanced self-care abilities of patients with CHF, including self-care maintenance, self-care management and self-care confidence (all P<0.05). The physical and mental quality of life was also significantly improved by CCM (P<0.01 or P<0.05). Compared with usual care, CCM significantly increased the LVEF (P<0.01), decreased the NT-proBNP level (P<0.01), and enhanced exercise capacity (P<0.001). In conclusion, CCM improved the self-care, quality of life and cardiac function of patients with CHF compared with usual care.

## Introduction

Heart failure is a final stage of heart disease, and it occurs when the heart muscle is unable to pump sufficient blood and oxygen to meet the needs of the body (1). Chronic heart failure (CHF) is a long-term condition of heart failure, which currently affects more than 23 million persons worldwide (2). CHF greatly decreases quality of life, reduces physical function, attention, executive function and memory (3). The symptoms of CHF commonly include weakness, fatigue, oliguria, memory loss, anxiety, headaches, insomnia, exertional dyspnea, orthopnea and edema (4). In many cases, behavioral modification, drug therapy and device treatment can significantly improve the outcomes (5). However, hospitalizations and readmissions for CHF remain major public health problems (6). The main goals of CHF treatment are the prolongation of life, and improvement of the quality of life.

The concept of collaborative care model (CCM) was developed to attempt to improve the quality of care (7). Over time, three core concepts, population-based care, measurement-based care and stepped care, are emphasized in CCM (8–11). In CCM, provision of care and health outcomes are defined based on a population of patients; each patient's progress is closely tracked using validated clinical rating scales (e.g., self-care index for heart failure); treatment is systematically adjusted, i.e., if patients do not improve as expected, initial adjustments can be made by the primary nurses (7,12). To date, multiple studies have tested the effects of CCM on different diseases, such as depression and schizophrenia, and CCM has been consistently regarded as an effective care mode (13,14). However, few reports have been focused on the interventional effects of CCM on CHF.

This study aimed to explore the effects of CCM on improving the care of patients with CHF.

## Material and Methods

### Study design and participants

A total of 114 CHF patients were enrolled in this study, from November 2013 to January 2016. All participants were randomly and equally divided into a control group and an experimental group (n=57 in each group). Patients had been diagnosed with CHF (systolic or diastolic dysfunction) with chest X-ray showing pulmonary edema, and New York Heart Association (NYHA) functional Class I-III. The left ventricular ejection fraction (LVEF) of patients in the two groups was lower than the normal range. Inclusion criteria for study participants were being older than 18 years old, and without dementia and acute coronary event in the prior 3 months. The spouse or the immediate family of these patients served as caregivers, and they accompanied the patients at the time of enrollment. This study was approved by the Sun Yat-Sen University Ethics Committee, and written informed consent was obtained from each adult before data collection.

Patients enrolled in this study were treated with spironolactone combined with metoprolol succinate. Spironolactone, a potassium-sparing diuretic, was used at a dose of 20 mg, three times a day. Metoprolol succinate (12.5-25 mg) was first used once a day, and then the dose was doubled every 2 weeks. If adverse effects were seen, including hypotension, fatigue, heart rate less than 55 bpm, fluid retention, II and III degree atrioventricular block, and myocardial infarction, dose doubling was delayed until the adverse effects disappeared.

### Intervention by usual care and CCM

Patients in the control group were treated with usual care, including management of their symptoms, diet, exercise, medication and psychology.

CCM was administrated to the experimental group, and was monitored by the caregivers and primary nurses. First, a primary nurse evaluated the self-care ability and psychological status of patients. The primary nurse worked collaboratively with patients and their caregivers to establish individualized clinical and self-care goals. Second, the primary nurse handed out a health education booklet and video-tape, and introduced the type, dosage, curative effects and adverse reactions of common drugs to patients and their caregivers. The primary nurse supervised patients and their caregivers to manage symptoms and lifestyles, including weight, blood pressure and heart rate measurement, water and salt intake, reasonable exercise and sufficient sleeping. Finally, caregivers thereafter carried out follow-up telephone calls at each week after hospital discharge to provide feedback from patients regarding their adherence to overall CCM (1).

### Self-care of heart failure index (SCHFI)

The SCHFI version 6.2 was carried out to measure self-care of CHF patients as described previously (1). SCHFI is widely used for measuring the self-care behaviors of CHF patient. It consists of three subscales, the self-care maintenance scale, the self-care management scale and the self-care confidence scale. The self-care maintenance scale has 10 items measuring self-monitoring and treatment adherence, such as checking weight and ankle swelling, taking medication regularly, low sodium intake and appropriate exercise. The self-care management scale has 6 items measuring the abilities of symptom recognition, treatment implementation and treatment evaluation, such as, when dyspnea or ankle swelling occur and how to implement remedies. The self-care confidence scale has 6 items measuring the confidence of patients, such as the confidence to manage CHF. All items used a 4-point self-report scale and each of these three self-care subscales was standardized from 0 to 100. Higher scores reflected better self-care.

### Short form health survey 12 (SF-12)

The SF-12 was used as previously described to measure the quality of life of CHF patients (15). It consists of 12 items and two dimensions, physical component summary and mental component summary. The score of physical component summary items measures physical quality of life, which is influenced by physical health, physical functioning, role limitations and bodily pain (16). The score of mental component summary items measures mental quality of life, which is influenced by mental health, vitality, role limitations and social functioning. Both dimensions were standardized from 0 to 100, and higher scores reflected better quality of life.

### LVEF measurement

LVEF of CHF patients was measured by standard echocardiographic examinations using a Sonos 5500 ultrasound machine (Philips, The Netherlands) with a 2.5-Hz transducer (17). The normal value of LVEF was >50%.

### N-terminal pro-B-type natriuretic peptide (NT-proBNP) measurement

NT-proBNP level was determined by a one-step sandwich enzyme-linked immunosorbent assay (ELISA) using the Novegent NT-proBNP ELISA kit (Chongqing Novegent Biotech Co., China), according to the manual (18). Before patients were transfused with packed red blood cells, 5 mL blood samples were collected into clot-tubes and used for measuring the NT-proBNP. Sera were extracted by centrifugation at 2500 *g* for 10 min at 4°C, and then stored at -80°C until analysis.

### 6-min walking test (6MWT)

The 6MWT is a simple method used for testing the exercise capacity of CHF patients. In brief, a marked hallway (30.5 m length) in the hospital was used to conduct the 6MWT. The patients were asked to walk back and forth along this hallway as far as possible for a period of 6 min, at their own best pace but not to run (19). Each patient had a personal instructor to provide encouragement and announce the remaining time. The total distance walked within 6 min was recorded, and normal exercise capacity was designated by a walking distance between 375 to 449.5 m.

### Statistical analysis

Data are reported as means±SD or as n (%), as appropriate. Statistical analysis was performed using SPSS software (version 17.0 for Windows; SPSS Inc., USA). The differences between the control and experimental groups were analyzed by independent *t*-test or chi-square test. The obtained estimations were considered statistically significant if P<0.05.

## Results

### Clinical and demographic characteristics

The mean patient age was 63.61±6.62 and 65.44±5.79 years in the control and CCM groups, respectively. Patients in the control and CCM groups had similar educational levels, and more than 75% of patients had less than high school education. The values of LVEF in control and CCM patients were 41.82±16.01 and 42.06±13.81%, respectively. In addition, patients were distributed throughout the four categories of NYHA functional class, although there was no class IV (symptomatic at rest) represented. Overall, there was no significant difference between the control group and CCM group in the mean age, gender, education, LVEF, and NYHA class, showing that the clinical and demographic characteristics of patients in the two groups were statistically equivalent (Table 1).

**Table 1.** Clinical and demographic characteristics of the control group (normal care, n = 57) and the collaborative care model (CCM) group (n=57) of chronic heart failure patients.

	Control patients	CCM patients	P
Age (years)	63.61±6.62	65.44±5.79	0.119
Gender			0.343
Male	36 (63.16%)	30 (52.63%)	
Female	21 (36.84%)	27 (47.37%)	
Education			0.809
Elementary	18 (31.58%)	17 (29.82%)	
Middle school	15 (26.32%)	16 (28.07%)	
Professional school	12 (21.05%)	16 (28.07%)	
High school	8 (14.04%)	6 (10.54%)	
University degree	4 (7.02%)	2 (3.51%)	
LVEF (%)	41.82±16.01	42.06±13.81	0.932
NYHA Class			0.747
I	11 (19.30%)	12 (21.05%)	
II	19 (33.33%)	22 (38.60%)	
III	27 (47.37%)	23 (40.35%)	
IV	0	0	

Data are reported as means±SD or number and percentages. CHF: chronic heart failure; LVEF: left ventricular ejection fraction measurement; NYHA: New York Heart Association. Statistical analysis was done with the *t*-test or chi-square test.

### CCM enhanced the self-care ability of CHF patients

**Figure 1.**
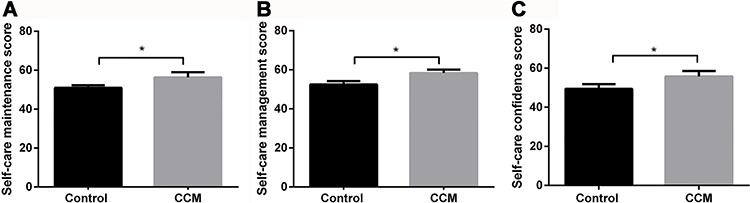
Comparison of *A*, self-care maintenance; *B*, self-care management, and *C*, self-care confidence between patients with chronic heart failure treated with usual care (Control) and with the collaborative care model (CCM). Data are reported as means±SD. *P<0.05 (*t*-test).

After 3 months of care, the SCHFI was performed to measure the self-care of CHF patients. The scores of self-care maintenance, management and confidence in CCM group were all significantly higher than the control group (all P<0.05; Figure 1). These results revealed that CCM enhanced the self-care ability of CHF patients.

### CCM enhanced the quality of life of CHF patients

**Figure 2.**
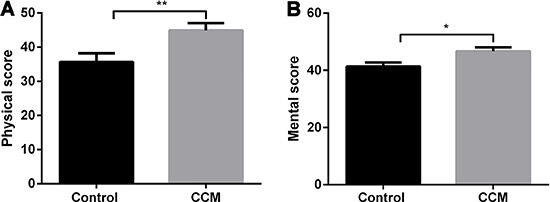
Comparison of *A*, physical quality of life, and *B*, mental quality of life between patients with chronic heart failure treated with usual care (Control) and with the collaborative care model (CCM). Data are reported as means±SD. *P<0.05; **P<0.01 (*t*-test).

CCM significantly improved both the physical and mental quality of life, compared with the control group (P*<*0.01 or P<0.05; Figure 2). Thus, we inferred that CCM also could improve the quality of life of CHF patients.

### CCM improved the cardiac function of CHF patients

**Figure 3.**
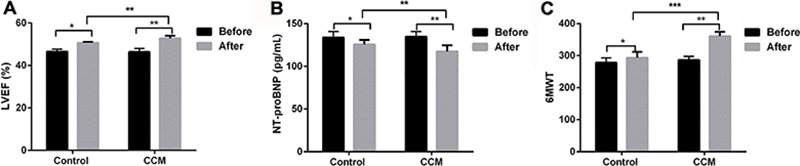
Comparison of *A*, left ventricular ejection fraction (LVEF); *B*, N-terminal pro-B-type natriuretic peptide (NT-proBNP) level, and *C*, exercise capacity between patients with chronic heart failure treated with usual care (Control) and with the collaborative care model (CCM). 6MWT: 6-min walking test. Data are reported as means±SD. *P<0.05; **P<0.01; ***P<0.001 (*t*-test).

As shown in Figure 3, both the usual care and CCM significantly increased the LVEF (P<0.05 or P<0.01), decreased the NT-proBNP level (P<0.05 or P<0.01), and enhanced the exercise capacity (P<0.05 or P<0.01), compared to baseline. More importantly, compared with usual care, CCM significantly increased the LVEF (P<0.01), decreased the NT-proBNP level (P<0.01), and enhanced the exercise capacity (P<0.001). Therefore, CCM improved the cardiac function of CHF patients.

## Discussion

CHF is a common chronic disease that requires much care. In the present study, we found that, compared with the control group, CCM significantly enhanced the self-care ability, i.e., self-care maintenance, self-care management and self-care confidence. More surprisingly, physical and mental quality of life was significantly enhanced, and cardiac function was greatly improved by CCM.

CCM is regarded as an effective and efficient integrated care mode in many chronic diseases (7). In human immunodeficiency virus (HIV) and other specialty physical health care settings, HIV Translating Initiatives for Depression into Effective Solutions (HITIDES) intervention serves as one type of CCM, and it improves depression and HIV symptom outcomes (20). In attention-deficit hyperactivity disorder (ADHD), CCM appeared feasible and acceptable for the treatment of Hispanic children with ADHD and showed preliminary evidence of effectiveness (21). In terms of CHF, CCM also showed to be effective, and was associated with increased use of drug therapies and cardiovascular diagnostic tests and better outcomes compared with primary care alone (22). Asch et al. (23) demonstrated that CCM improved a wide range of processes of care for CHF, including medical therapeutics, and education and counseling. Consistent with these previous studies, our findings showed that CCM could significantly improve self-care, quality of life and cardiac function of patients with CHF.

Self-care is defined as an individual's capacity to perform health promotion, disease prevention or maintenance, and even a capacity to address specific behaviors by changing the sense of well-being (24). It is essential to improve outcomes of patients with CHF (25,26). Several studies demonstrated that, collaboration with visiting nurses or a combination of telemonitoring counseling effectively promoted self-care, and then reduced readmission rates and promoted health (27–29). Katon et al. (30) reported that the self-care ability of patients with depression was enhanced by CCM with education encompassing self-monitoring, behavioral activation, goal setting and problem solving. Consistently, we found that CCM was essential for improving self-care abilities and outcomes in patients with CHF.

Quality of life and cardiac function, together with self-care, are three important issues in CHF (16). Poor quality of life and cardiac function always accompany all stages of CHF. In addition, quality of life serves as a pivotal outcome measurement in any evaluation of treatment efficacy (31). Yehle et al. (32) found that with telemonitoring and home healthcare nurse visiting quality of life was remarkably improved. Moreover, exercising regularly has been found to be associated with major improvements in the quality of life and cardiac function (33,34). Although the precise molecular mechanism(s) of action remain unclear, physical exercise has been advocated as a non-pharmacological adjuvant to strengthen the conventional management and prevention of cardiovascular events (35). A series of clinical and experimental studies has demonstrated that an appropriate volume and intensity of exercise can ameliorate myocardial dysfunction through the improvement of maximum oxygen consumption, LVEF, LV diastolic and systolic volumes, ventilatory threshold, cardiac output and diastolic function (E/A ratio) (35–38). In the present study, the patients in the CCM group received telemonitoring from primary nurses and exercise training, which might partly explain the ameliorative effects of CCM on quality of life and cardiac function. Besides, the influence of caregivers' emotion on behavior of patients might contribute to these ameliorative effects. In one study of 27 patients with CHF and their family caregivers, a positive orientation toward solving problems in caregivers was a significant predictor of improved quality of life in patients (39).

As a limitation, the patients enrolled in this study had mild CHF, as evidenced by preserved LVEF values, low NT-proBNP values, and no NYHA functional class IV represented.

In conclusion, the current data showed that CCM improved the self-care, quality of life and cardiac function of patients with mild CHF when compared with usual care. Whether CCM would also improve NYHA class IV patients still needs to be investigated. Further research is also needed to understand the underlying mechanism of this effect.
